# Two Accessory Proteins Govern MmpL3 Mycolic Acid Transport in Mycobacteria

**DOI:** 10.1128/mBio.00850-19

**Published:** 2019-06-25

**Authors:** Allison Fay, Nadine Czudnochowski, Jeremy M. Rock, Jeffrey R. Johnson, Nevan J. Krogan, Oren Rosenberg, Michael S. Glickman

**Affiliations:** a Immunology Program, Sloan Kettering Institute, New York, New York, USA; b Division of Infectious Diseases, Memorial Sloan Kettering Cancer Center, New York, New York, USA; c Immunology and Microbial Pathogenesis Graduate Program, Weill Cornell Graduate School, New York, New York, USA; d Program for Microbial Pathogenesis, University of California, San Francisco, San Francisco, California, USA; e Division of Infectious Diseases, Department of Medicine, University of California, San Francisco, San Francisco, California, USA; f Chan-Zuckerberg Biohub, San Francisco, California, USA; g Laboratory of Host-Pathogen Biology, The Rockefeller University, New York, New York, USA; h Department of Cellular and Molecular Pharmacology, University of California, San Francisco, San Francisco, California, USA; i Quantitative Biosciences Institute (QBI), University of California, San Francisco, San Francisco, California, USA; j The J. David Gladstone Institutes, San Francisco, California, USA; New York University School of Medicine

**Keywords:** *Mycobacterium*, *Mycobacterium tuberculosis*, cell envelope, transporters

## Abstract

Mycolic acids are the signature lipid of mycobacteria and constitute an important physical component of the cell wall, a target of mycobacterium-specific antibiotics and a mediator of Mycobacterium tuberculosis pathogenesis. Mycolic acids are synthesized in the cytoplasm and are thought to be transported to the cell wall as a trehalose ester by the MmpL3 transporter, an antibiotic target for M. tuberculosis. However, the mechanism by which mycolate synthesis is coupled to transport, and the full MmpL3 transport machinery, is unknown. Here, we identify two new components of the MmpL3 transport machinery in mycobacteria. The protein encoded by *MSMEG_0736*/*Rv0383c* is essential for growth of Mycobacterium smegmatis and M. tuberculosis and is anchored to the cytoplasmic membrane, physically interacts with and colocalizes with MmpL3 in growing cells, and is required for trehalose monomycolate (TMM) transport to the cell wall. In light of these findings, we propose MSMEG_0736/Rv0383c be named “TMM transport factor A”, TtfA. The protein encoded by *MSMEG_5308* also interacts with the MmpL3 complex but is nonessential for growth or TMM transport. However, MSMEG_5308 accumulates with inhibition of MmpL3-mediated TMM transport and stabilizes the MmpL3/TtfA complex, indicating that it may stabilize the transport system during stress. These studies identify two new components of the mycobacterial mycolate transport machinery, an emerging antibiotic target in M. tuberculosis.

## Abstract (precis)

The cell envelope of Mycobacterium tuberculosis, the bacterium that causes the disease tuberculosis, is a complex structure composed of abundant lipids and glycolipids, including the signature lipid of these bacteria, mycolic acids. In this study, we identified two new components of the transport machinery that constructs this complex cell wall. These two accessory proteins are in a complex with the MmpL3 transporter. One of these proteins, TtfA, is required for mycolic acid transport and cell viability, whereas the other stabilizes the MmpL3 complex. These studies identify two new components of the essential cell envelope biosynthetic machinery in mycobacteria.

## INTRODUCTION

Mycobacteria have a complex cell wall, which is crucial for maintaining cell integrity, protects against environmental stress, provides a barrier against access of potentially harmful molecules into the cell, and plays a critical role in mycobacterial pathogenesis. The cell wall of mycobacteria is comprised of the common bacterial cell wall glycopolymer, peptidoglycan, external to the cytoplasmic membrane, as well as an additional covalently attached glycopolymer layer comprised of arabinogalactan. Arabinogalactan bridges peptidoglycan and mycolic acids, the signature long-chain lipid of mycobacteria. Arabinogalactan-esterified mycolates likely constitute the inner leaflet of the outer membrane bilayer, with the outer leaflet comprising hydrophobically associated complex lipids, including trehalose dimycolate, sulfolipids, lipomannan, and lipoarabinomannan. This outer membrane increases both the complexity of the cell wall structure and its hydrophobicity. The enzymatic steps of the arabinogalactan and mycolate precursor biosynthesis have been well described and are the targets of several antimycobacterials, including isoniazid and ethambutol (1–3).

Mycolic acid synthesis begins with the fatty acid synthase I (FASI) system that produces C_16_ to C_18_ and C_24_ to C_26_ fatty acids. The FASII system then extends these products to produce the long meromycolate chains that are the substrates for the polyketide synthetase, Pks13. Pks13 catalyzes the final condensation step to produce α-alkyl β-ketoacids (C_60_ to C_90_) which are then acetylated and transferred to the 6 position of trehalose (4, 5). Mono-α-alkyl β-ketoacyl trehalose is then reduced by CmrA to trehalose monomycolate (TMM) presumably in the inner leaflet of the cytoplasmic membrane (6, 65). TMM can then be modified by nonessential mycolic acid methyltransferases to produce cyclopropane rings and methyl branches, and in the case of Mycobacterium tuberculosis, these modifications alter host-mycolic acid responses (8–14).

After synthesis, TMM must be transported across the cytoplasmic membrane to reach the cell wall; this step is known to require the MmpL3 transporter (25–27). The MmpLs (mycobacterial membrane protein, large) are multisubstrate transporters of the resistance-nodulation-division (RND) class that usually act as homotrimers and are exporters of molecules from the outer leaflet of the plasma membrane to or through the outer membrane. In M. tuberculosis, they include lipid and fatty acid transporters that transport virulence-associated lipids across the cell envelope. Transport is driven by downhill movement of H^+^ in response to the electrochemical H^+^ gradient ($\Delta {\tilde{\text{μ}}}_{{\text{H}}^{+}}$) across the plasma membrane. MmpL3 is the only MmpL protein that is essential *in vitro*, though mutations in several other MmpLs severely compromise virulence in infection models (7, 15–18). Mutational analyses and “transposon-site hybridization” (TraSH) revealed MmpL3 is essential for M. tuberculosis viability *in vitro* (19) and *in vivo* (20), and several inhibitors of MmpL3 are already in clinical development, among them are a set of diamine-indole-carboxamides (21–23), including Novartis NITD-304 and the pyrrole BM212 (24).

Genetic, pharmacologic, and biochemical studies strongly indicate that the MmpL3 transporter is the TMM flippase. MmpL3 has been shown to have flippase activity in spheroplast assays (25), and genetic depletion leads to growth arrest and loss of TMM transport (26, 27). Recent crystal structures of MmpL3 suggest potential mechanisms of TMM transport (28). However, the full mechanisms linking TMM biosynthesis to MmpL3 transport, and the full set of components used by MmpL3 to transport TMM, are unknown.

MmpLs share homology with other bacterial RND proteins, typified by the acridine resistance complex (AcrB) transporter that is involved in the efflux of hydrophobic small molecules from or through the periplasm of Escherichia coli. Like AcrB, the MmpLs are thought to be localized to the inner membrane (29). However, AcrB does not act alone: it additionally forms the core of a comprehensive secretion system that traverses both the inner and outer membranes of the cell envelope in Gram-negative bacteria, allowing the AcrB substrates to bypass the periplasm (30). To form this membrane-spanning system, AcrB interacts with a periplasmic coupling protein called AcrA (or more generally, the membrane fusion protein [MPF]), which in turn links to an outer membrane channel called TolC (or more generally, the outer membrane protein [OMP]) (31–33). The mechanism of bacterial RND transporters is thought to be highly conserved and involves the engagement of the proton motive force ($\Delta {\tilde{\text{μ}}}_{{\text{H}}^{+}}$) to drive drugs, ions, and other small molecules from the periplasm across the outer membrane through the MPF, thus preventing the entrance of toxic substances into the bacterial cytoplasm (34–36). However, no identifiable AcrA or TolC homologues are evident in mycobacterial genomes, raising the question of whether MmpL3 acts in concert with other proteins. Here, we describe two previously unknown components of the MmpL3 complex, one of which is required for TMM transport and one of which is stress inducible and stabilizes the MmpL3 complex.

## RESULTS

### MmpL3 is stably associated with two proteins of unknown function, MSMEG_0736 and MSMEG_5308.

**FIG 1:**
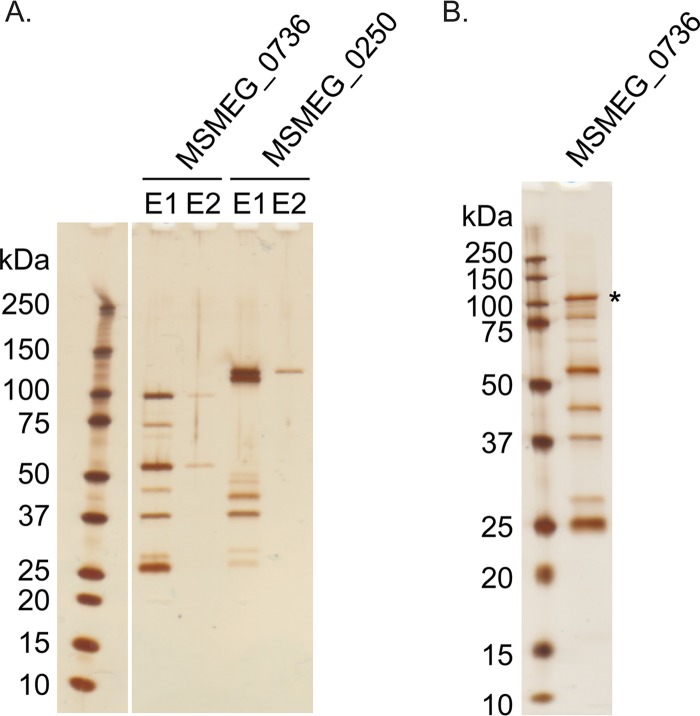
MmpL3 and MSMEG_0736 form a complex. (A) Silver-stained SDS-PAGE gel of elutions from GFP-Trap purifications using detergent solubilized membranes from M. smegmatis expressing MSMEG_0736-msfGFP (MGM6425) or MmpL3-msfGFP (MSMEG_0250 [MGM6464]). See Table 1 for protein identifications. (B) Silver-stained SDS-PAGE gel of the first elution from a GFP-Trap purification using detergent solubilized membranes from M. smegmatis expressing MSMEG_0736-msfGFP. The band corresponding to the molecular weight of MmpL3, indicated with an asterisk, was excised and subjected to mass spectrometry analysis and identified as MmpL3 (see Materials and Methods and Table S2 in the supplemental material).

To discover stable binding interactions with Mycobacterium smegmatis MmpL3 (MsMmpL3) *in situ*, we devised a native, stringent affinity purification. MsMmpL3 was fused to a flexible linker connecting the C terminus of MmpL3 to monomeric superfolder green fluorescent protein (msfGFP) at the native chromosomal locus of MmpL3. As *mmpL3* is an essential gene, the normal growth rate of this strain suggests that fusion did not disrupt the essential function of the protein. Cell membranes were collected and solubilized with the mild detergent *n*-dodecyl β-d-maltoside (DDM). Anti-GFP nanobodies covalently linked to a magnetic bead were incubated with detergent-solubilized membranes and then extensively washed with 0.2% DDM-containing buffer. Copurified proteins were identified via shotgun mass spectrometry (Fig. 1, Table 1). One of the most abundantly copurified proteins was a protein of unknown function, MSMEG_0736 (Table 1; see also Tables S1A and S1B in the supplemental material). In a control experiment using MmpL10 (MSMEG_0410) fused to msfGFP as a bait, neither MSMEG_0736, MmpL3, nor MSMEG_5308 copurified (Tables S1A and S1B). To validate this interaction, we created a strain in which an msfGFP was fused to MSMEG_0736. When MSMEG_0736-msfGFP was purified from detergent-solubilized membranes under the same conditions, the most abundantly copurified protein was MmpL3 (Tables 1, S1A, and S1B). In a biological replicate of the MSMEG_0736 pulldown, we confirmed the identity of the prominent band at approximately 100 kDa as MmpL3 (Fig. 1B; see also Table S2). As MSMEG_0736 interacts with MmpL3, and evidence we will present in this paper shows MSMEG_0736 is required for TMM transport, we propose MSMEG_0736 be named “TMM transport factor A,” TtfA.

**TABLE 1:** MSMEG_0736 and MSMEG_0250 protein-protein interactions

Bait	ProtID	Name	No. of unique peptides^*a*^	Gene name
MSMEG_0736	A0QP27	MSMEG_0250	35	*MmpL3*
	A0QQF4	MSMEG_0736	22	*ttfA*
	A0R316	MSMEG_5308	14	*MSMEG_5308*
MSMEG_0250 (MmpL3)	A0QP27	MSMEG_0250	57	*MmpL3*
	A0QQF4	MSMEG_0736	11	*ttfA*
	A0R316	MSMEG_5308	15	*MSMEG_5308*

a Number of unique peptides detected in the GFP-Trap eluates using MSMEG_0736-msfGFP or MSMEG_0250-msfGFP as bait. Only the three proteins with the highest number of unique peptides are shown.

10.1128/mBio.00850-19.6 TABLE S1 Identification of proteins interacting with MSMEG_0736 (TtfA), MSMEG_0250 (MmpL3), or MSMEG_0410 (MmpL10). Download Table S1, XLSX file, 0.1 MB. Copyright © 2019 Fay et al. 2019 Fay et al. This content is distributed under the terms of the Creative Commons Attribution 4.0 International license.

10.1128/mBio.00850-19.7 TABLE S2 Protein identification by mass spectrometry of excised band from MSMEG_0736 (TtfA)-msfGFP GFP-Trap pulldown denoted by asterisk in Fig. 1B. Download Table S2, XLSX file, 0.1 MB. Copyright © 2019 Fay et al. 2019 Fay et al. This content is distributed under the terms of the Creative Commons Attribution 4.0 International license.

Analysis of MsTtfA and MsMmpL3 copurifying proteins identified by anti-GFP nanobody purification showed a third complex member found in both pulldowns, the protein encoded by *MSMEG_5308*. This seven-bladed beta-propeller protein has a homolog in M. tuberculosis, Rv1057, which has been shown to be nonessential, although M. tuberculosis lacking Rv1057 fails to properly secrete ESAT-6 and replicated poorly in macrophages (37). The Rv1057 gene has been shown to be under the control of two two-component systems involved in sensing cell stress, MprAB and TcrRS, as well as the envelope stress-responsive sigma factor SigE (38–40). Rv1057 was also reported to be the most transcriptionally induced gene in response to MmpL3 depletion (41), suggesting a connection to MmpL3 function.

### TtfA is essential for growth of M. smegmatis and M. tuberculosis *in vitro*.

**FIG 2:**
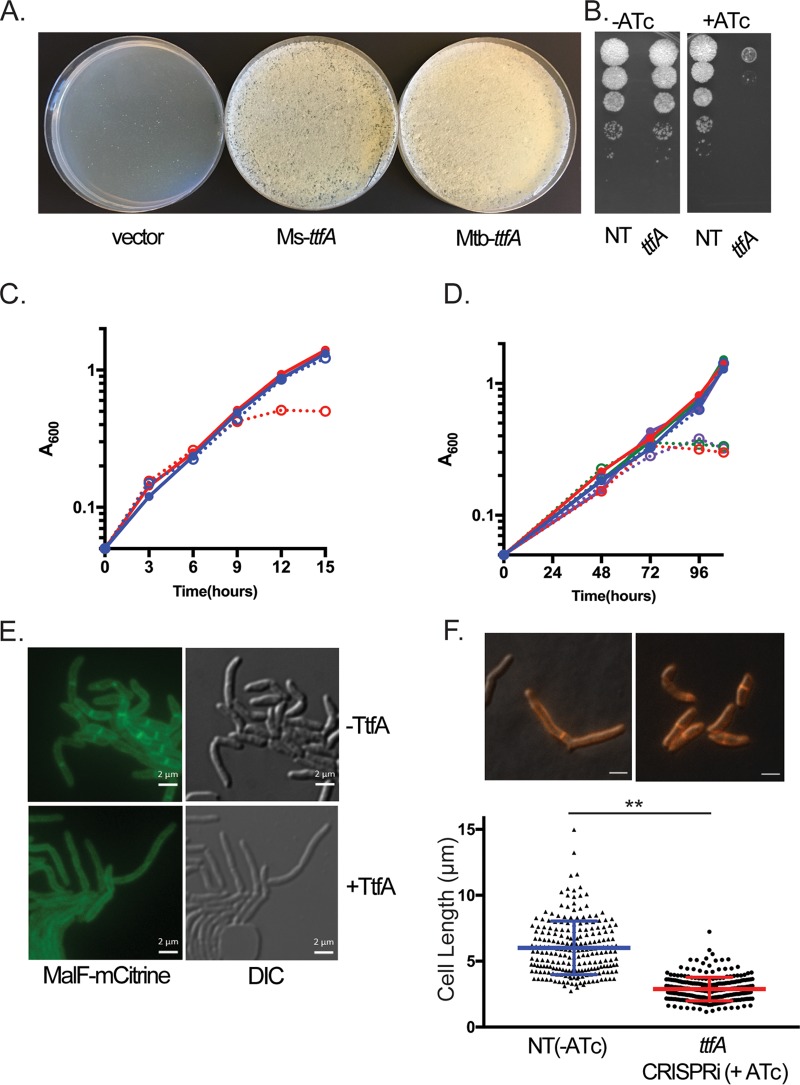
MsTtfA/MtbTtfA is required for mycobacterial growth and cell elongation. (A) M. smegmatis strains with deletion of chromosomal *ttfA* and carrying a copy of *ttfA* at the *attB* phage integration site were subjected to marker exchange with *attB* integrating vectors. Δ*ttfa attB*::*ttfA strep* (MGM6414) strains transformed with pMV306kan (vector), pAJF792 (encoding MsTtfA), or pAJF793 (MtbTtfA) are shown on kanamycin agar. (B) Ten-fold dilutions of M. smegmatis carrying ATc-inducible CRISPRi nontargeting control (NT [MGM6418]) or *ttfA* (MGM6419) on agar media with and without ATc. (C) Growth curves of nontargeting (MGM6418, blue) and *ttfA*-targeting (MGM6419, red) CRISPRi M. smegmatis strains grown under uninduced (solid, closed circles) and ATc-induced (dashed, empty circles) conditions. (D) Growth curves of nontargeting (MGM6715, blue) or three distinct M. tuberculosis *ttfA*-targeting CRISPRi M. tuberculosis strains (MGM6675, red; MGM6677, green; MGM6679, purple) grown under uninduced (solid, closed circles) and ATc-induced (dashed, empty circles) conditions. (E) Fluorescence microscopy of an M. smegmatis *ttfA*-targeting CRISPRi strain marked with MalF(1,2)-mCitrine (MGM6433) 15 h post-CRISPRi induction with ATc (top, −TtfA) or, an uninduced control at 15 h (+TtfA, bottom). YFP (left) and DIC (right) images shown. Exposure times for YFP, 250 ms, 40% LED. (F) Loss of TtfA leads to short cells. Cell lengths of nontargeting (MGM6418, triangles) and TtfA-targeting (MGM6419, circles) CRISPRi strains induced for 12 h. Representative DIC/FM 4-64 images used for quantitation shown above the graph. Error bars are standard deviation and ** indicates *P* < 0.001.

The M. tuberculosis H37Rv homolog of TtfA is Rv0383c. *rv0383c* was predicted to be an essential gene in H37Rv based on transposon mutagenesis (19, 42), but its essentiality in M. smegmatis and M. tuberculosis is unknown and its molecular function obscure. With no predicted protein domains or homologs of known function, confirmation of its essentiality in both organisms was the first step to analyze its function. To test the essentiality of *ttfA* in M. smegmatis, we generated a merodiploid strain in which a second copy of *ttfA* was integrated in the chromosome. We then deleted the endogenous coding sequence, so that the only a single copy of *ttfA* remained at the *attB* site. We then attempted to remove the second copy of *ttfA* from *attB* by marker exchange with either a vector or a plasmid encoding TtfA and conferring kanamycin resistance, pAJF792 (43). Only transformation with the plasmid encoding TtfA yielded transformants that were kanamycin resistant and streptomycin sensitive. Similar results were obtained with a plasmid encoding TtfA from M. tuberculosis (Fig. 2A). This inability to remove *ttfA* from *attB* in our Δ*ttfA* strain suggested that *ttfA* was required for growth of M. smegmatis (Fig. 2A). To further assess the essential role of MsTtfA, we generated a CRISPR interference (CRISPRi) strain that allows anhydrotetracycline (ATc) inducible knockdown (44). Growth inhibition by gene knockdown was visualized by spotting 10-fold serial dilutions on plates with and without ATc, and MsTtfA depletion led to an ATc-dependent growth defect not seen in the nontargeting control (Fig. 2B). Gene knockdown of *ttfA* in M. smegmatis also led to cessation of growth in liquid medium between 9 and 12 h postinduction with ATc (Fig. 2C). To test whether TtfA was essential in M. tuberculosis, we attempted to knockout the gene using a temperature-sensitive phage and were unsuccessful, suggesting essentiality. We then generated three *ttfA*-targeting CRISPRi strains with independent guide RNAs. Gene knockdown of *ttfA* in M. tuberculosis with all three guide RNAs led to cessation of growth in liquid medium after 3 days after induction with ATc, indicating that TtfA is essential for M. tuberculosis growth *in vitro* (Fig. 2D).

To examine the morphologic changes that accompany growth arrest during loss of MsTtfA, we depleted the protein using CRISPRi and tracked morphological changes using a MalF(1,2)-mCitrine expression strain that uniformly labels the cell membrane (43). Time-lapse microscopy indicated that growth arrest without MsTtfA was characterized by shorter misshapen cells (Fig. 2E; see also Movies S1 and S2). Quantitation of cell length revealed that MsTtfA-depleted cells were significantly shorter (2.88 ± 0.89 μm) than control cells (6.00 ± 2.03 μm) (Fig. 2F). The short cell phenotype suggested that MsTtfA might be required for cell elongation. These data indicate that TtfA is essential for mycobacterial viability and that the function of this gene is conserved between fast and slow growing mycobacteria.

10.1128/mBio.00850-19.9 MOVIE S1 Time-lapse microscopy of MGM6433, MalF(1,2)-mCitrine *ttfA*-targeting CRISPRi strain, grown in microfluidic plate under uninduced conditions. Imaged every hour for 16 h. YFP image shown. Exposure time, 250 ms. Bar, 2 μm. Download Movie S1, AVI file, 15.0 MB. Copyright © 2019 Fay et al. 2019 Fay et al. This content is distributed under the terms of the Creative Commons Attribution 4.0 International license.

10.1128/mBio.00850-19.10 MOVIE S2 Time-lapse microscopy of MGM6433, MalF(1,2)-mCitrine Msmeg_0736-targeting CRISPRi strain, grown in microfluidic plate and induced with ATc 250 ng/ml after 1 h. Imaged every hour for 16 h. YFP image shown. Exposure time, 250 ms. Bar, 2 μm. Download Movie S2, AVI file, 15.0 MB. Copyright © 2019 Fay et al. 2019 Fay et al. This content is distributed under the terms of the Creative Commons Attribution 4.0 International license.

### TtfA localizes to poles and septa.

The predicted protein for MsTtfA contains a predicted N-terminal transmembrane domain from amino acids 2 to 24, indicating that it is either a transmembrane or secreted protein. To determine the localization and topology of MsTtfA, we assessed the functionality of mCitrine fused at the N or C terminus. Marker exchange with a plasmid encoding MsTtfA-mCitrine yielded kanamycin-resistant streptomycin-sensitive transformants in similar numbers to that with pAJF792, encoding the wild-type protein indicating the C-terminal fusion is functional. In contrast, the plasmid encoding an N-terminal mCitrine fusion did not yield kanamycin-resistant streptomycin-sensitive transformants, indicating that this fusion failed to complement for essential function.

**FIG 3:**
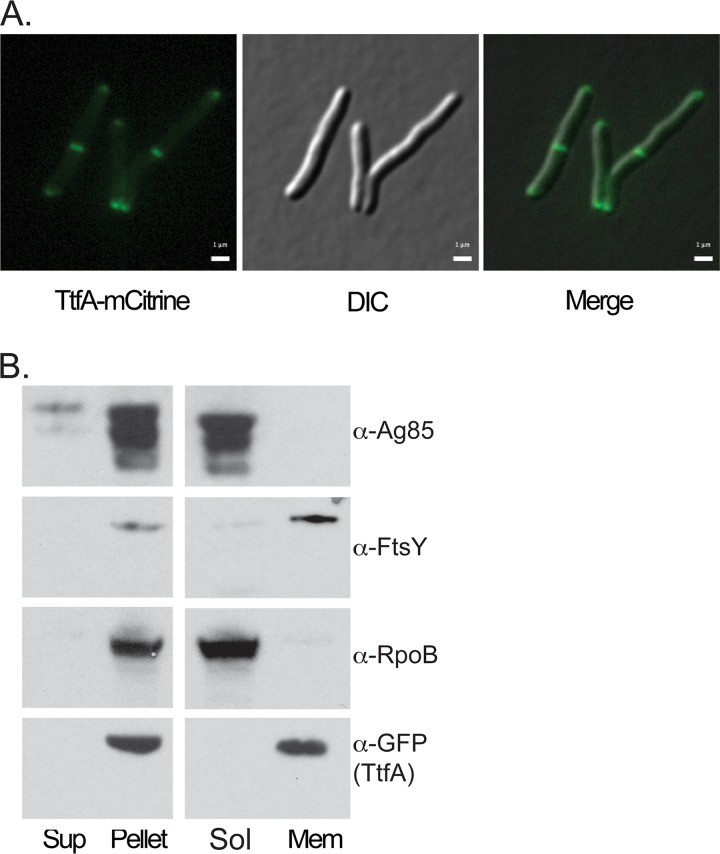
TtfA is a membrane protein that localizes to poles and septa. (A) M. smegmatis TtfA-mCitrine expression strain (MGM6423) imaged during logarithmic growth. YFP (left), DIC (middle), and overlay (right) images shown. Bars, 1 μm. Exposure time for YFP, 1 s, 75% LED. (B) Localization of TtfA-msfGFP by cellular fractionation. Cell-free supernatant and cell pellet fractions (left) and soluble and membrane fractions (right) probed for secreted protein Ag85 (top), membrane protein FtsY (top middle), cytoplasmic protein RpoB (bottom middle), and GFP for TtfA-msfGFP (bottom).

We next localized MsTtfA using live-cell fluorescence microscopy. The C-terminal mCitrine fusion protein produced fluorescent signals at the cell poles and septa (Fig. 3A; see also Movie S3, available at https://www.dropbox.com/sh/vspwtjjd5151jo1/AADXuv-jeL7qSXJpN8m8WU3Ra?dl=0). It was previously reported that mCitrine does not fluoresce when localized in the periplasm, suggesting that the C-terminal domain of MsTtfA is localized in the cytoplasmic side of the membrane (45). We then generated an MsTtfA C-terminal fusion to msfGFP by recombination, such that the fused copy was expressed from its endogenous locus and was the only copy, guaranteeing functionality. The resulting MsTtfA-msfGFP strain demonstrated fluorescent signals exclusively at cell poles and septa (see Movie S4, available at the URL mentioned above). Fractionation of cell-free supernatants showed no detectable MsTtfA-msfGFP in the supernatant (Fig. 3B), suggesting that the protein is not secreted. Fractionation of the cell lysate showed that MsTtfA-msfGFP localized in the Triton-X-100-soluble fraction, similarly to a membrane protein control FtsY but not the soluble fraction marked by cytosolic RNAPβ, supporting that MsTtfA is membrane anchored, is not secreted, and has a cytoplasmic C terminus.

### The essential portion of TtfA is conserved among mycolate producers.

To further delineate the functional domains of the protein, we examined the conservation of the protein sequence across homologs. BLAST searches identified homologous predicted proteins among mycolate-producing organisms (see Fig. S1). Alignments of these homologs suggested that amino acids 1 through approximately 205 were well conserved, with poor conservation in the C-terminal 73 amino acids (Fig. S1). The C-terminal 73 amino acids are also predicted to be disordered (46). This lack of conservation at the C terminus was also apparent in the alignment with the M. tuberculosis TtfA (MtbTtfA), which we demonstrated as described above is functional in M. smegmatis (Fig. 2A). To assess the functional contribution of these conserved regions, we generated MsTtfA truncations fused at the C terminus to msfGFP and assessed the ability of these truncations to complement the essential function by marker exchange. Only the plasmid encoding amino acids 1 to 205 yielded kanamycin-resistant streptomycin-sensitive transformants, indicating that amino acids 1 to 205 were essential (see Fig. S2A).

10.1128/mBio.00850-19.1 FIG S1 (A) MUSCLE alignment of 16 reciprocal BLAST hits of TtfA. Aligned residues shown in green. Residue ruler shown for TtfA below TtfA sequence and for the overall alignment above the alignment. (B) Alignment tree of 94 TtfA reciprocal BLAST hits. Tree based on MUSCLE alignment. Download FIG S1, PDF file, 0.8 MB. Copyright © 2019 Fay et al. 2019 Fay et al. This content is distributed under the terms of the Creative Commons Attribution 4.0 International license.

10.1128/mBio.00850-19.2 FIG S2 Accumulation and localization of TtfA truncations. (A) Immunoblot of strains expressing full-length or truncations of MSMEG_0736 fused to msfGFP. Lysates of TtfA-msfGFP (MGM6829) and TtfA-msfGFP truncations (1-23 [MGM6826], 1-50 [MGM6823], 1-100 [MGM6827], 1-150 [MGM6824], 1-205 [MGM6822], 24-278 [MGM6825]). Blots probed with for GFP (top) or RNAPβ (bottom) as loading control. (B) Localization of TtfA truncations fused to GFP. Residues of TtfA fused shown to left of image. TtfA-msfGFP truncations (1-23 [MGM6826], 1-50 [MGM6823], 1-100: [MGM6827], 1-150 [MGM6824], 1-205 [MGM6822], 24-278 [MGM6825]). GFP and GFP/DIC overlay shown. Exposure times for GFP, 500 ms, 75% LED. Download FIG S2, PDF file, 0.4 MB. Copyright © 2019 Fay et al. 2019 Fay et al. This content is distributed under the terms of the Creative Commons Attribution 4.0 International license.

After confirming that all of these truncations accumulate as stable proteins at their predicted sizes when expressed in wild-type M. smegmatis (Fig. S2A), we localized each truncation by fluorescence microscopy. MsTtfA(1-205aa)-msfGFP localized to poles and septa in a pattern similar to that of the full-length protein (Fig. S2B), indicating that the poorly conserved C terminus is not required for essential function or proper localization. However, truncations shorter than 205 amino acids (aa), which did not complement essential function, also failed to localize to poles and septa, indicating that the first 205 aa of the protein, including the N-terminal transmembrane domain, are required for proper localization and that this localization is tightly linked to its essential function.

### The N terminus of TtfA is required for interaction with MmpL3.

**FIG 4:**
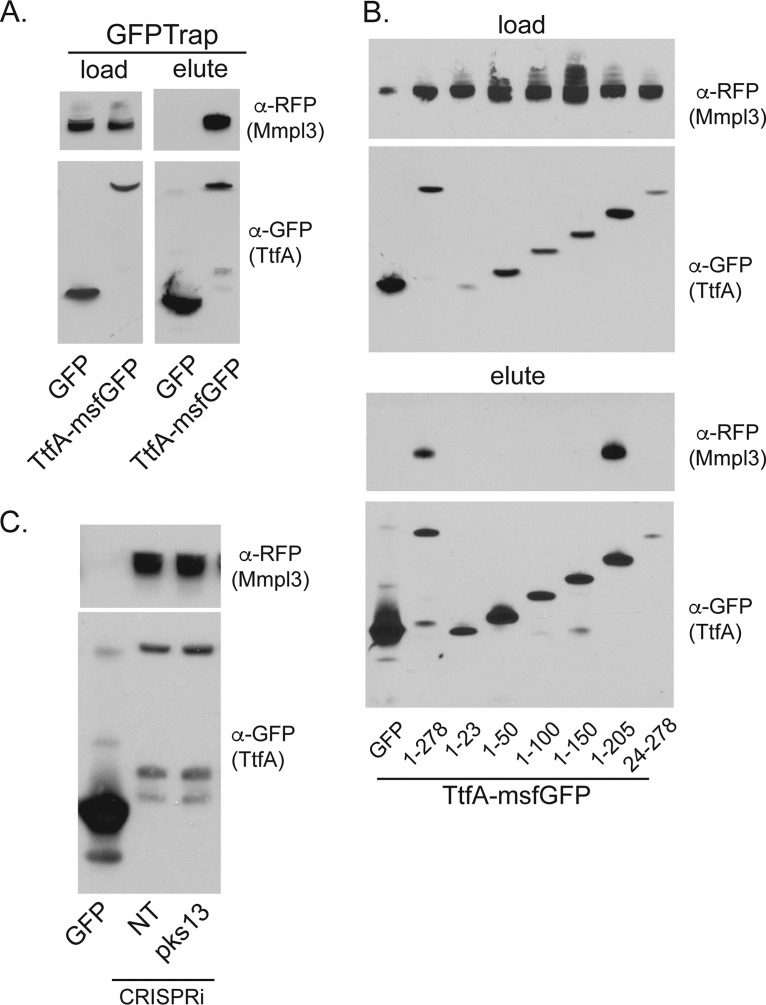
TtfA and MmpL3 form a complex *in vivo* via the essential region of TtfA and independently of TMM synthesis. (A) DDM-solubilized M. smegmatis lysates (left) and GFP-Trap eluates (right) of msfGFP-expressing control (MGM6828) and TtfA-msfGFP (MGM6815) both coexpressing MmpL3-mCherry and probed with anti-RFP (top) and anti-GFP (bottom). (B) The essential region of MsTtfA is necessary and sufficient for MmpL3 interaction. DDM-solubilized lysates (top) and GFP-Trap eluates (bottom) of msfGFP control (MGM6828), full-length TtfA-msfGFP (1-278 [MGM6829]), or TtfA-msfGFP truncations (1-23 [MGM6826], 1-50 [MGM6823], 1-100 [MGM6827], 1-150 [MGM6824], 1-205 [MGM6822], 24-278 [MGM6825]) coexpressing MmpL3-mCherry and probed with anti-RFP (top) and anti-GFP (bottom). (C) The MsTtfA-MmpL3 interaction is independent of mycolate synthesis. GFP-Trap eluates of MmpL3-mCherry expression strains coexpressing msfGFP control (MGM6828) or TtfA-msfGFP with either control CRISPRi (NT [MGM6816]) or Pks13 depleted (MGM6817) for 6 h with ATc. Top panel is probed for MmpL3-mCherry with anti-RFP and bottom with anti-GFP.

To determine the regions of MsTtfA required for interaction with MmpL3, we immunopurified MsTtfA truncations fused to msfGFP when coexpressed with MmpL3-mCherry. MsTtfA-msfGFP was purified from DDM detergent-solubilized lysates with GFP-Trap resin. Unfused msfGFP did not coprecipitate MmpL3-mCherry, whereas full-length MsTtfA-msfGFP copurified with MmpL3-mCherry (Fig. 4A). All truncations were visible in DDM-solubilized lysates at their predicted sizes (Fig. 4B). However, only MsTtfA(1-205aa)-msfGFP copurified with MmpL3-mCherry, quantitatively similar to full-length MsTtfA-msfGFP (Fig. 4B). However, loss of any segment of MsTtfA within the first 205 aa abolished interaction with MmpL3. These results demonstrate an exact correlation between the ability of MsTtfA to interact with MmpL3 and the essential function of this protein, suggesting that the essentiality of TtfA may be due to a role as an MmpL3 complex component.

### The MmpL3 and MSMEG_0736 complex and its localization are independent of TMM biosynthesis.

To examine whether the TtfA-MmpL3 interaction requires TMM synthesis, the substrate of the MmpL3 flippase, we depleted Pks13, the TMM synthetase in M. smegmatis (47, 48). Depletion of Pks13 in the MmpL3-mCherry/TtfA-msfGFP strain caused growth arrest after 6 h of induction, indicative of the depletion of essential Pks13 (see Fig. S3). However, Pks13 depletion did not affect levels of either TtfA-msfGFP or MmpL3-mCherry in DDM-solubilized lysates (data not shown) and had no effect on the TtfA-msfGFP-MmpL3-mCherry complex (Fig. 4C). These results indicate that active TMM biosynthesis is not required for TtfA-MmpL3 complex formation.

10.1128/mBio.00850-19.3 FIG S3 Pks13 depletion leads to growth arrest by 9 h after CRISPRi induction. Growth curves of *pks13*-targeting (MGM6745) CRISPRi strain grown with no ATc (blue, circles) or with ATc (red, squares). Download FIG S3, PDF file, 0.3 MB. Copyright © 2019 Fay et al. 2019 Fay et al. This content is distributed under the terms of the Creative Commons Attribution 4.0 International license.

**FIG 5:**
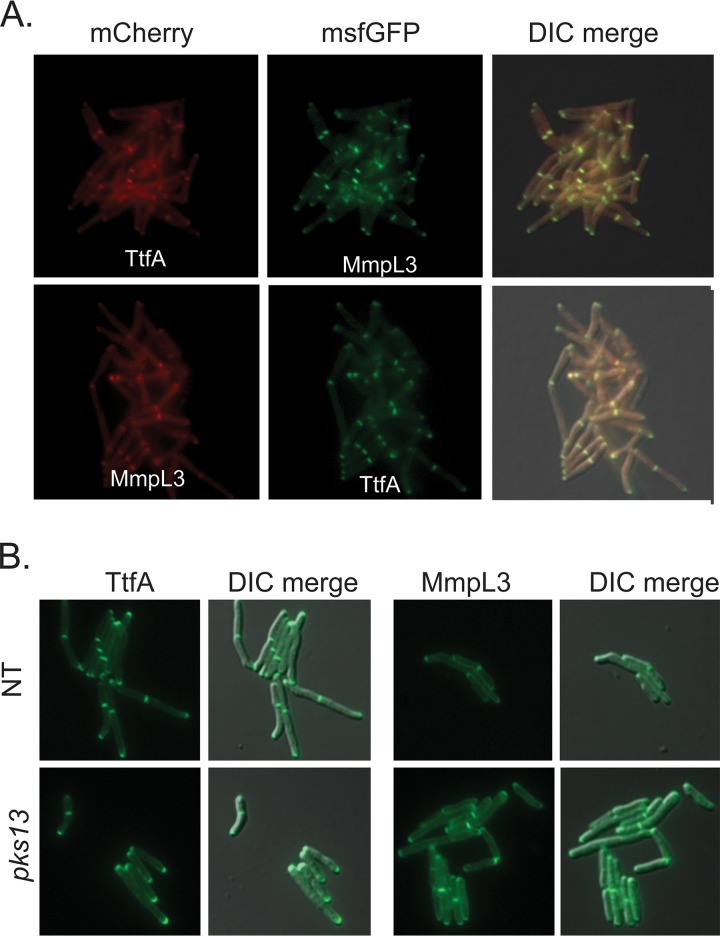
TtfA and MmpL3 colocalize at cell poles and septa independently of TMM synthesis. (A) Localization of MsTtfA-mCherry/MmpL3-msfGFP (MGM6433, top) and MmpL3-mCherry/TtfA-msfGFP (MGM6434, bottom). (B) Localization of TtfA-msfGFP or MmpL3-msfGFP in Pks13-depleted or mock-depleted cells.

MmpL3-GFP was previously reported to localize to cell poles and septa (49), a finding we confirmed with our MmpL3-msfGFP strain, which localizes the MmpL3 protein to poles and septa (see Movie S5, available at https://www.dropbox.com/sh/vspwtjjd5151jo1/AADXuv-jeL7qSXJpN8m8WU3Ra?dl=0). This pattern is very similar to the pattern observed with TtfA-msfGFP (Movie S4). To colocalize MmpL3 and TtfA we again utilized strains coexpressing mCherry and msfGFP fusions to TtfA and MmpL3. By fluorescence microscopy, MmpL3 and TtfA strongly colocalized to cell poles and septa (Fig. 5A) and were indistinguishable in their localization patterns. Depletion of Pks13 via CRISPRi led to cessation of growth between 6 and 9 h but did not affect localization of TtfA-msfGFP or MmpL3-msfGFP, again indicating that TMM synthesis was not required for localization of either protein to the poles or septa (Fig. 5B). Taken together, these results strongly indicate that MmpL3 and TtfA form a complex *in vivo* at the site of cell growth.

### TtfA is required for MmpL3 TMM transport in M. smegmatis and M. tuberculosis.

**FIG 6:**
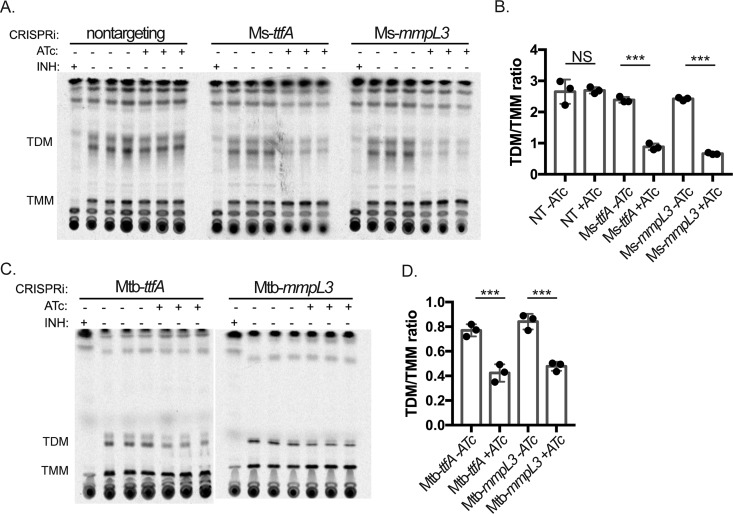
MsTtfA and MtbTtfA are required for TMM transport. (A) Thin-layer chromatographs (TLCs) of extractable mycolic acids from three replicate ^14^C-acetic acid-labeled M. smegmatis cultures carrying CRISPRi targeting guide RNAs (nontargeting [MGM6418]; left), *ttfA* (MGM6419; middle), or *mmpL3* (MGM6637; right). Isoniazid treatment served as a positive control for inhibition of all mycolate synthesis. (B) Graph of TDM/TMM ratios for quantitation of TMM and TDM of TLCs in panel A. (C) TLCs of extractable mycolic acids from three replicate ^14^C-acetic acid-labeled M. tuberculosis cultures depleted for TtfA (MGM6675; left) or MmpL3 (MGM6676; right) (D) Quantitation of TDM/TMM ratios from quantitation of TMM and TDM of TLCs in panel C. ***, *P* < 0.01.

We next assessed whether TtfA is functionally required for TMM flipping. Loss of MmpL3 function via genetic or pharmacologic inhibition results in TMM accumulation and trehalose dimycolate (TDM) depletion due to the inability of TMM to flip across the cytoplasmic membrane where antigen 85 enzymes process TMM to TDM and arabinogalactan attached mycolic acids (25–27). To assess the functional role of TtfA in TMM flipping, we utilized CRISPRi strains with depleted TtfA or MmpL3 and a nontargeting control in M. smegmatis. Six hours after knockdown of gene expression, we labeled mycolic acids with ^14^C-acetate and assessed TMM/TDM levels in cell wall organic extracts. Depletion of MmpL3 had the reported effect of ^14^C-TMM accumulation and ^14^C-TDM depletion (Fig. 6A), attributable to MmpL3 dysfunction. Depletion of TtfA had a quantitatively similar effect on TMM transport as depletion of MmpL3, as shown by the TDM/TMM ratio in depleted cultures compared to that in replete cultures (Fig. 6A and B). As a control for essential protein depletion, we depleted the essential DnaK chaperone (43) and found no effect on ^14^C-TMM/^14^C-TDM, indicating that cell arrest by depletion of any essential protein does not alter TMM and TDM levels (see Fig. S4).

10.1128/mBio.00850-19.4 FIG S4 DnaK depletion does not impact TMM and TDM levels. (A) TLCs of extractable mycolic acids from ^14^C-acetic acid-labeled cultures of MGM6005, replete (lanes 2 to 4) or depleted of DnaK for 16 h (lanes 5 to 7) prior to labeling for 1 h with ^14^C-acetic acid. Lane 1 is an INH-treated replete control. (B) Graph of TDM/TMM ratios for quantitation of TMM and TDM of TLCs in panel A. Download FIG S4, PDF file, 0.4 MB. Copyright © 2019 Fay et al. 2019 Fay et al. This content is distributed under the terms of the Creative Commons Attribution 4.0 International license.

We saw similar results in M. tuberculosis depleted of TtfA or MmpL3. Depletion of either TtfA or MmpL3 impaired TMM transport, with the resulting accumulation of TMM and loss of TDM (Fig. 6C and D). These results indicate that loss of the MmpL3-interacting protein TtfA impairs MmpL3-dependent TMM transport in both M. smegmatis and M. tuberculosis, strongly indicating that TtfA is an essential cofactor in MmpL3 function.

### An additional complex member is responsive to MmpL3 and TtfA depletion and inhibition of flippase activity.

**FIG 7:**
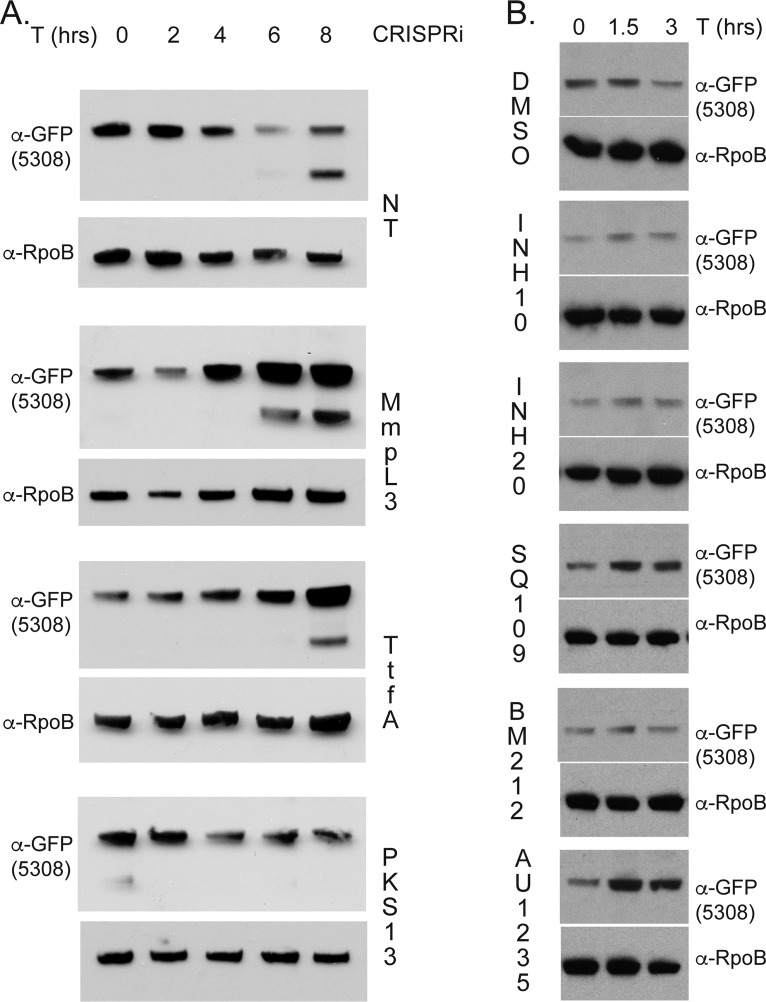
MSMEG_5308-msfGFP accumulates in response to MmpL3 dysfunction. (A) Lysates of MSMEG_5308-msfGFP expression strains with CRISPRi constructs nontargeting control (NT [MGM6766]), MmpL3 (MGM6718), TtfA (MGM6717), or Pks13 (MGM6767) (ATc induction at 0, 2, 4, 6, and 8 hrs) and probed with anti-GFP or anti-RpoB. Anti-GFP blot shown between 75 kDa and 63 kDa as determined by molecular weight marker. (B) Lysates of MSMEG_5308-msfGFP expression strain (MGM6681) treated with dimethyl sulfoxide [DMSO], 10 μg/ml INH, 20 μg/ml INH, 5 μM SQ109, 5 μM BM212, or 5 μM AU1235 for 0, 1.5, or 3 hrs and probed with anti-GFP and anti-RpoB (loading control).

MSMEG_5308 was also found to copurify with both MsTtfA and MmpL3 (Fig. 1 and Table 1). To further investigate this MmpL3 complex member, we generated a C-terminal msfGFP fusion to MSMEG_5308 at the chromosomal locus. We then depleted either MmpL3 or TtfA in the MSMEG_5308-msfGFP strain. Depletion of either MmpL3 or TtfA, but not the nontargeting control, led to accumulation of MSMEG_5308 protein (Fig. 7A). In contrast, CRISPRi depletion of Pks13 led to cessation of cell growth after 6 h of induction but did not induce MSMEG_5308 accumulation (Fig. 7A).

We further examined the response of MSMEG_5308 to inhibitors of the TMM/TDM pathway, including early mycolate biosynthesis (isoniazid [INH]) and inhibitors targeting late steps in TMM/TDM transport (SQ109, BM212, and AU1235). Resistance mutations to SQ109, BM212, and AU1235 arise in MmpL3; however, only BM212 and AU1235 have been shown to directly inhibit MmpL3 flippase activity, and all three inhibitors may have effects outside MmpL3 flippase activity (24–26, 50). Both SQ109 and AU1235 caused MSMEG_5308-msfGFP accumulation at 1.5 and 3 h, but INH or BM212 (at 5 and 10 μM) had no effect (Fig. 7B and data not shown). The lack of accumulation of MSMEG_5308 with INH treatment or Pks13 depletion suggests that MSMEG_5308 does not accumulate in response to loss of TMM or TDM biosynthesis but rather accumulates in response to inhibition of their transport.

### MSMEG_5308-msfGFP localizes to cell poles and septa and stabilizes MmpL3/TtfA interaction.

**FIG 8:**
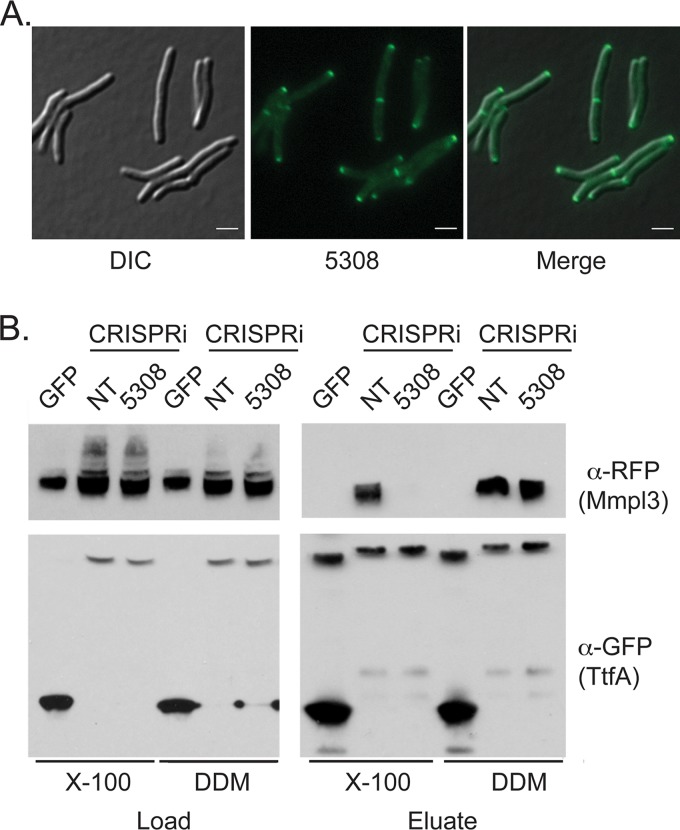
MSMEG_5308 localizes to cell poles and septa and stabilizes the TtfA/MmpL3 interaction. (A) MSMEG_5308-msfGFP expression strain (MGM6681) imaged during logarithmic growth. GFP, DIC, and merged images are shown. (B) GFP-Trap pulldown of TtfA-msfGFP/MmpL3-mCherry coexpression strains with nontargeting or MSMEG_5308-targeting CRISPRi constructs. Inputs (left) and eluates (right) from the GFP-Trap column in the presence of either DDM or Triton X-100.

The identification of MSMEG_5308 as an MmpL3/TtfA interacting protein suggested that MSMEG_5308 may colocalize with the MmpL3 complex. Indeed, MSMEG_5308-msfGFP localized to cell poles and septa in a pattern similar to those of both TtfA-msfGFP and MmpL3-msfGFP by live cell fluorescence microscopy (Fig. 8A). To examine the role of MSMEG_5308, we targeted *MSMEG_5308* using CRISPRi and verified efficient knockdown using an MSMEG_5308-msfGFP strain (see Fig. S5). Depletion of MSMEG_5308 had no impact on growth or cell morphology, confirming MSMEG_5308 was not essential in M. smegmatis (data not shown).

10.1128/mBio.00850-19.5 FIG S5 MSMEG_5308-msfGFP depletion using CRISPRi. Lysates from MGM6730 grown without or with ATc for 12 h and probed for GFP (top) or or RNAPβ (bottom) as loading control. Download FIG S5, PDF file, 0.5 MB. Copyright © 2019 Fay et al. 2019 Fay et al. This content is distributed under the terms of the Creative Commons Attribution 4.0 International license.

To assess the effect of MSMEG_5308 on MmpL3/TtfA complexes, we isolated TtfA-msfGFP using anti-GFP nanobodies and probed for MmpL3-mCherry in the presence and absence of MSMEG_5308. In DDM-solubilized lysates, TtfA copurified with MmpL3-mCherry in MSMEG_5308-depleted lysates similarly to control cells (Fig. 8B). However, in Triton X-100-solubilized lysates, although the MmpL3-TtfA complex was intact when MSMEG_5308 was present, TtfA-msfGFP did not coprecipitate MmpL3-mCherry in the absence of MSMEG_5308 (Fig. 8B). These results indicate that MSMEG_5308 is a nonessential member of the MmpL3 complex that is induced by stress and stabilizes the MmpL3-TtfA protein complex.

## DISCUSSION

We have identified two new components of the essential machinery of mycolic acid transport and cell growth in mycobacteria. The MmpL3 transporter was previously known to transport trehalose monomycolate, but its cofactors were unknown. The MmpL3 machinery contains the essential protein TtfA, which we show is required for TMM flipping across the cytoplasmic membrane. A third complex member, MSMEG_5308, while not required for TMM transport, appears to stabilize the MmpL3 complex and is upregulated in response to MmpL3 dysfunction. All three of these proteins localize to cell poles and septa, which are the sites of cell elongation and the previously reported localization sites of early mycolic acid synthetic machinery such as MabA and InhA as well as MmpL3 (49).

TtfA, a protein with no predicted domains of known function, is an essential component of the mycolic acid transport machinery. We have defined the essential portions of TtfA, amino acids 1 to 205 that include the N-terminal transmembrane domain but not the poorly conserved disordered C terminus. Using coprecipitation techniques, we see that truncations that disrupt localization and interaction with MmpL3 fail to support the essential TMM transport function of MmpL3. Our model for the molecular function of TtfA in TMM transport is that the protein links the mycolate biosynthetic machinery to the MmpL3 transporter, possibly by binding directly to TMM. MmpL3 is distinct from several other MmpL proteins in that disruption of the transporter does not inhibit synthesis of the transported lipid. For several MmpL proteins, transport and synthesis are linked. For example, deletion of the sulfolipid transporter MmpL8 abolishes sulfolipid synthesis rather than simply inhibiting its transport (16, 51). Similarly, MmpL7 is physically and functionally coupled to phthiocerol dimycocerosate (PDIM) biosynthesis (52). Additionally, loss of MmpL10 abolishes synthesis of polyacyltrehaloses and trehalose polyphleates (53–55). However, the lack of such coupling in the MmpL3 system may suggest that a coupling protein is required to chaperone the transported glycolipid to the transporter, a function we hypothesize for TtfA.

Alternatively, it is possible that TtfA is a scaffolding protein that nucleates additional essential MmpL3 complex members yet to be elucidated. TtfA was previously shown to interact with the nonessential vesiculogenesis regulator VirR in M. tuberculosis, which we also find in our purifications of MsTtfA (56).

The second protein we identify in the MmpL3 complex, MSMEG_5308, is a seven-bladed propeller protein. This protein structural motif was previously described to aid in protein-protein interactions, though members are functionally diverse (57–59). In M. tuberculosis, the MSMEG_5308 homolog, Rv1057, is responsive to a variety of membrane stresses as well as MmpL3 depletion (38, 39, 41). Our data indicate that the function of MSMEG_5308 is to stabilize the MmpL3/TtfA complex. We hypothesize that MSMEG_5308 is upregulated during times of membrane stress in order to stabilize MmpL3 complexes and preserve TMM transport and cell wall biosynthesis under conditions that may dissociate the MmpL3 complex.

MmpL3-mediated TMM transport has emerged as an attractive drug target after several high-throughput screens identified whole-cell active inhibitors that appear to target this transporter. Our identification of two previously unidentified cofactors for MmpL3 will empower future studies to investigate these proteins as drug targets and their potential roles in cellular response and resistance to MmpL3-targeting small molecules. Additionally, future biochemical and structural studies will examine the biochemical and structural organization of this essential mycolic acid transport complex.

## MATERIALS AND METHODS

### Bacterial and DNA manipulations.

Standard procedures were used to manipulate recombinant DNA and to transform E. coli. M. smegmatis strains were derivatives of mc^2^155 (43). M. tuberculosis strains are derivatives of Erdman. Gene deletions were made by homologous recombination and double negative selection (43). All strains, plasmids, and CRISPRi oligonucleotides used in this study are listed in Table S3 in the supplemental material. M. smegmatis and M. tuberculosis were transformed by electroporation (2,500 V, 2.5 μF, 1,000 Ω). All M. smegmatis strains were cultured in LB or 7H9 medium with 0.5% glycerol, 0.5% dextrose (LBsmeg/7H9smeg). M. tuberculosis was grown in 7H9-oleic acid-albumin-dextrose-catalase (OADC); 0.05% Tween 80 was added to all liquid media. Antibiotic concentrations used for selection of M. smegmatis and M. tuberculosis strains were as follows: kanamycin, 20 μg/ml,; hygromycin, 50 μg/ml; streptomycin, 20 μg/ml. For CRISPRi knockdowns, anhydrotetracycline (ATc) was used at 50 ng/ml (M. smegmatis) or 100 ng/ml (M. tuberculosis).

10.1128/mBio.00850-19.8 TABLE S3 Bacterial strains, plasmids, and CRISPRi oligonucleotides used in this study. Download Table S3, XLSX file, 0.1 MB. Copyright © 2019 Fay et al. 2019 Fay et al. This content is distributed under the terms of the Creative Commons Attribution 4.0 International license.

### Immunoblotting.

For protein and epitope tag detection, GFP (rabbit anti-GFP polyclonal antibody, 1 mg/ml, 1:20,000; Rockland Immunochemicals), mCherry (rabbit anti-RFP polyclonal antibody, 1 mg/ml, 1:20,000; Rockland Immunochemicals), RNAP-β (8RB13 mouse anti-E. coli RNAPβ monoclonal, 1:20,000; Neoclone), and Ag85 (rabbit polyclonal antibody, 1:20,000; BEI Resources) antibodies were used.

### Microscopy.

All images were acquired using a Zeiss Axio Observer Z1 microscope equipped with Definite focus, stage top incubator (Insert P Lab-Tek S1, TempModule S1), Colibri2.0 and Illuminator HXP 120 C light sources, a Hamamatsu ORCA-Flash4.0 CMOS camera, and a Plan-Apochromat 100×/1.4 numerical aperture oil differential inference contrast (DIC) lens objective. Zeiss Zen software was used for acquisition and image export. The following filter sets and light sources were used for imaging: GFP (38 HE, Colibri2.0 470 light-emitting diode [LED]), mCherry (64 HE, Colibri2.0 590 LED), yellow fluorescent protein (YFP) (46 HE, Colibri2.0 505 LED) and FM 4-64 (20, HXP 120 C). For cell staining, 100 μl of culture was used. A final concentration of 1 μg/ml FM 4-64 (Invitrogen) was added. Cells were pelleted by centrifugation at 5,000 × *g* for 1 min and resuspended in 50 μl of medium. For single-time-point live-cell imaging, 7 μl of culture was spotted onto a number 1.5 coverslip and pressed to a slide. For time-lapse microscopy, cells were added to a 1.5% low-melting-point agarose LBsmeg pad. For pad preparation, LBsmeg agarose was heated to 65°C and poured into a 17-mm by 28-mm Gene Frame (AB-0578; Thermo Scientific) adhered to a 25-mm by 75-mm glass slide. A second slide was pressed down on top, and the setup was allowed to cool at room temperature for 10 min. The top slide was removed, and the pad was cut and removed so that a 3- to 4-mm strip remained near the center. Two to three microliters of M. smegmatis culture was added to the pad, and a number 1.5 24-mm by 40-mm cover glass was sealed to the Gene Frame. Slides were incubated in a stage-top incubator at 37°C. For time-lapse microscopy, cells were incubated in CellAsic ONIX microfluidic system (plates for bacterial cell culture, B04A) at a flow of 2.0 psi and heated at 37°C. Cells were equilibrated in plates at 37°C for 3 h prior to the start of imaging. Cell lengths were quantitated using Zeiss Zen software.

### ^14^C-Acetic acid labeling and TLC.

M. smegmatis and M. tuberculosis cultures were grown and depleted for the following times: M. smegmatis CRISPRi, 6 h in ATc 50 ng/ml; M. smegmatis Tet-DnaK, 16 h without ATc; and M. tuberculosis CRISPRi, 26 h in ATc 100 ng/ml. For TMM and TDM labeling, 1 ml of culture was removed and labeled for 1 h with 1 μl for M. smegmatis or 16 h with 2 μl for M. tuberculosis using [1-^14^C]acetic acid, sodium salt (1 mCi/ml, NEC084H001MC; Perkin Elmer). For INH controls, 20 μg/ml INH was added 5 min prior to label addition. After the incubation, cells were harvested by centrifugation at 10,000 × *g* for 5 min, and the supernatant was removed. The pellet was resuspended in 500 μl chloroform-methanol (2:1) and incubated at 37°C for 2 h. Cells and debris were pelleted at 10,000 × g for 5 min, and the supernatant was removed. Ten microliters of chloroform-methanol extraction was spotted on high-performance thin layer chromatography (HPTLC) plates, run 3 times in chloroform-methanol-water (90:10:1), allowed to air dry, and then imaged using a Phosphor storage cassette and Typhoon Trio (pixel size, 200 μm at best sensitivity). ImageJ64 was used to quantitate the radioactive signal.

### Protein expression and purification.

Endogenous MSMEG_0736 (TtfA), MSMEG_0250 (MmpL3), or MSMEG_0410 (MmpL10) was purified from M. smegmatis mc^2^155 expressing native MSMEG_0736 (MGM6425), MSMEG_0250 (MGM6464), or MSMEG_0410 (MGM6484) with a C-terminal msfGFP-tag. M. smegmatis strains were grown in 7H9 with 0.05% (vol/vol) Tween 80. Harvested cells were washed three times with phosphate-buffered saline (PBS) and frozen before lysis with a cryogenic grinder (SPEX SamplePrep). The powder was resuspended in 50 mM Tris-HCl (pH 7.5), 150 mM NaCl, SIGMAFast protease inhibitor cocktail (Sigma-Aldrich), and 0.6 to 0.7 units/ml Benzonase endonuclease, and the solution was incubated for 30 min. Solutions were centrifuged at 15,000 × g for 30 min, followed by centrifugation at 98,000 × g to 99,594 × g (depending on amount of material) for 1 h to isolate the membranes. Membranes were solubilized for 1 h at 4°C in 50 mM Tris-HCl (pH 7.5), 150 mM NaCl, and 1% DDM using a 1:0.10 (wt/wt) ratio of membranes to detergent. MSMEG_0410 was solubilized using a 1:0.15 (wt/wt) ratio of membranes to detergent. The solutions were centrifuged for 30 min at 99,526 × g to 103,530 × g. Solubilized membranes were incubated with GFP-Trap_MA beads (ChromoTek) for 1 h at 4°C. The beads were washed three times with 50 mM Tris-HCl (pH 7.5), 150 mM NaCl, and 0.2% DDM. Proteins were eluted from the beads by the addition of 0.2 M glycine (pH 2.5), and the eluate was neutralized with 1 M Tris base (pH 10.4). The elution was repeated a second time.

### Mass spectrometry.

The two GFP-Trap_MA elutions were pooled, and proteins were precipitated with trichloroacetic acid. The pellets were resuspended in 0.1% RapiGest SF in 50 mM ammonium bicarbonate. Samples were prepared for mass spectrometry analysis as previously described (60). Samples were denatured and reduced in a buffer containing 2 M urea and 2 mM dithiothreitol (DTT). Free cysteines were alkylated by the addition of 2 mM iodoacetamide. The reduced and alkylated samples were then digested with trypsin overnight at 37°C. Digested samples were desalted using UltraMicroSpin C_18_ columns (Nest Group) and then evaporated to dryness. Samples were resuspended in 0.1% formic acid for mass spectrometry analysis.

Samples were analyzed on a Thermo Scientific Orbitrap Fusion mass spectrometry system equipped with an Easy nLC 1200 ultrahigh-pressure liquid chromatography system interfaced via a nanoelectrospray source. Samples were injected onto a C_18_ reverse phase capillary column (75-μm inner diameter by 25-cm length, packed with 1.9-μm C_18_ particles). Peptides were then separated by an organic gradient from 5% to 30% acetonitrile (ACN) in 0.1% formic acid over 180 min at a flow rate of 300 nl/min. The mass spectrometer (MS) continuously collected spectra in a data-dependent fashion over the entire gradient.

Raw mass spectrometry data were analyzed using the MaxQuant software package (version 1.3.0.5) (61). Data were matched to the Mycobacterium smegmatis UniProt reference proteome database. Variable modifications were allowed for methionine oxidation and protein N terminus acetylation. A fixed modification was indicated for cysteine carbamidomethylation. Full trypsin specificity was required. The first search was performed with a mass accuracy of ±20 ppm, and the main search was performed with a mass accuracy of ±6 ppm. A maximum of 5 modifications were allowed per peptide. A maximum of 2 missed cleavages were allowed. The maximum charge allowed was 7+. Individual peptide mass tolerances were allowed. For MS/MS matching, a mass tolerance of 0.5 Da was allowed, and the top 6 peaks per 100 Da were analyzed. MS/MS matching was allowed for higher charge states and water and ammonia loss events. Data were searched against a concatenated database containing all sequences in both forward and reverse directions, with reverse hits indicating the false discovery rate of identifications. The data were filtered to obtain a peptide, protein, and site-level false discovery rate of 0.01. The minimum peptide length was 7 amino acids.

Protein identification from a single SDS-PAGE band was performed by the Taplin Mass Spectrometry Facility at Harvard Medical School. The gel band corresponding to the molecular weight of MmpL3 was excised from the Coomassie-stained gel. Excised gel bands were cut into approximately 1-mm^3^ pieces and subjected to a modified in-gel trypsin digestion procedure as follows (62). Gel pieces were washed and dehydrated with acetonitrile for 10 min, followed by removal of acetonitrile. Pieces were then completely dried in a SpeedVac. The gel pieces were rehydrated with a 50 mM ammonium bicarbonate solution containing 12.5 ng/μl modified sequencing-grade trypsin (Promega, Madison, WI) at 4°C. After 45 min, the excess trypsin solution was removed and replaced with 50 mM ammonium bicarbonate solution to just cover the gel pieces. Samples were then placed in a 37°C room overnight. Peptides were later extracted by removing the ammonium bicarbonate solution, followed by one wash with a solution containing 50% acetonitrile and 1% formic acid. The extracts were then dried in a SpeedVac (∼1 h). The samples were then stored at 4°C until analysis.

On the day of analysis, the samples were reconstituted in 5 to 10 μl of high-pressure liquid chromatography (HPLC) solvent A (2.5% acetonitrile, 0.1% formic acid). A nanoscale reverse-phase HPLC capillary column was created by packing 2.6-μm C_18_ spherical silica beads into a fused silica capillary (100-μm inner diameter by ∼30-cm length) with a flame-drawn tip (63). After equilibrating the column, each sample was loaded via a Famos auto sampler (LC Packings, San Francisco CA) onto the column. A gradient was formed and peptides were eluted with increasing concentrations of solvent B (97.5% acetonitrile, 0.1% formic acid).

As peptides eluted, they were subjected to electrospray ionization and then entered into an LTQ Orbitrap Velos Pro ion-trap mass spectrometer (Thermo Fisher Scientific, Waltham, MA). Peptides were detected, isolated, and fragmented to produce a tandem mass spectrum of specific fragment ions for each peptide. Peptide sequences (and hence protein identity) were determined by matching protein databases with the acquired fragmentation pattern by the software program, Sequest (Thermo Fisher Scientific, Waltham, MA) (64). All databases include a reversed version of all the sequences. The data were filtered and the resulting peptide false discovery rate was zero.

### GFP-Trap pulldown of MsTtfA truncations and MsTtfA-msfGFP with CRISPRi depletion.

Fifty milliliters of LBsmeg cultures of TtfA-msfGFP truncations and MmpL3-mCherry coexpression strains with CRISPRi targeting constructs were grown to an OD_600_ of 0.5. Nontargeting and MSMEG_5308 depletion strains were grown with ATc 50 ng/ml for 24 h, and Pks13 depletion strains were grown with ATc for 6 h. Cultures were cooled on ice, and cells were harvested by centrifugation (3,700 × g for 10 min at 4°C). Pellets were washed once with 1 ml of PBS. Pellets were resuspended in 500 μl PBS with 1× HALT protease (Thermo Scientific) and lysed via bead beating (Mini-beadbeater-16; Biospec) 2 times for 1 min with 5 min on ice between. Beads, unbroken cells, and debris were pelleted at 5,000 × g for 10 min at 4°C. The supernatant was collected, and an additional 500 μl of PBS containing either 1% DDM or 1% Triton X-100 was added and incubated at 4°C for 1 h with rocking. Insoluble material was then pelleted at 21,130 × g for 1 h at 4°C, and the supernatant (∼1 ml) was collected and added to 20 μl prewashed GFP-Trap magnetic agarose beads (Bulldog Bio) and incubated for 2 h at 4°C with rocking. After the incubation, the beads were collected with a magnet and washed 3 times with 1 ml PBS and 0.1% DDM or Triton X-100. Elution was performed using SDS sample buffer with heating at 60°C for 15 min.

### Data availability.

Data for mass spectrometry identification of interacting proteins are provided in Table S1A and B.
