# Supplementary figures and images for: Two Accessory Proteins Govern MmpL3 Mycolic Acid Transport in Mycobacteria
Source: mBio. 2019 Jun 25;10(3):e00850-19. doi: 10.1128/mBio.00850-19 (PMC6593404; doi:10.1128/mBio.00850-19)

A.

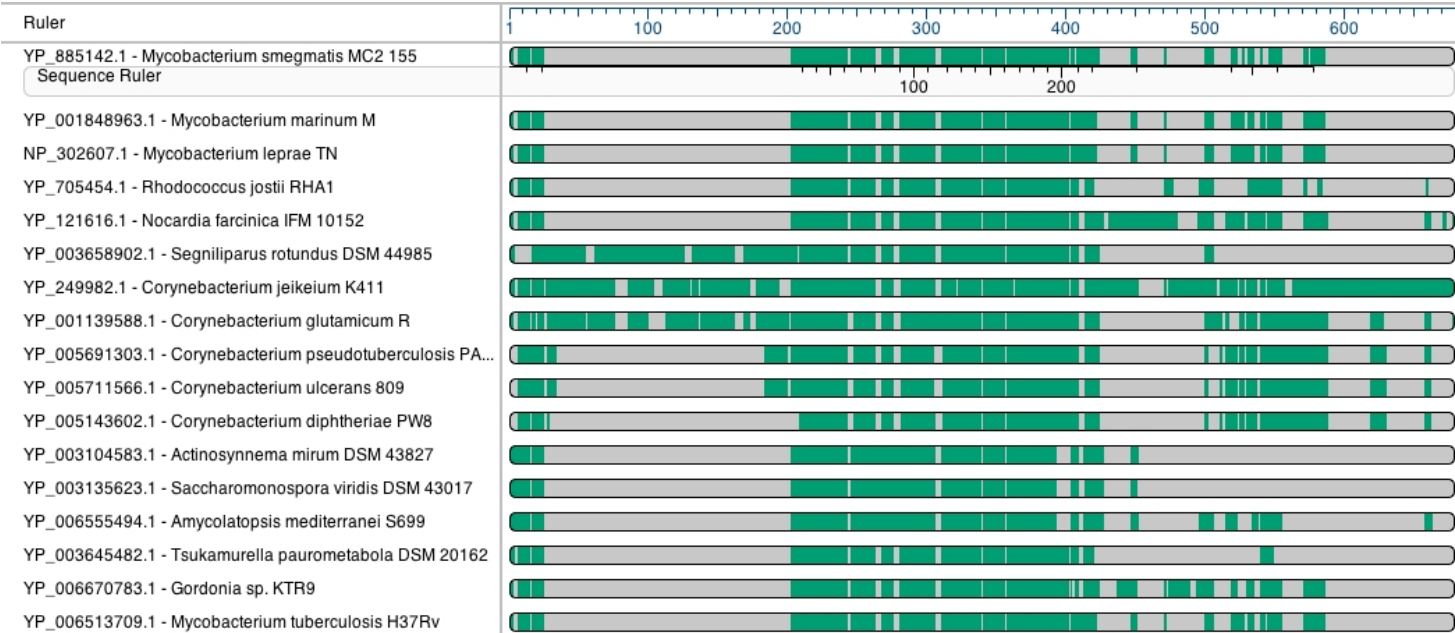

B.

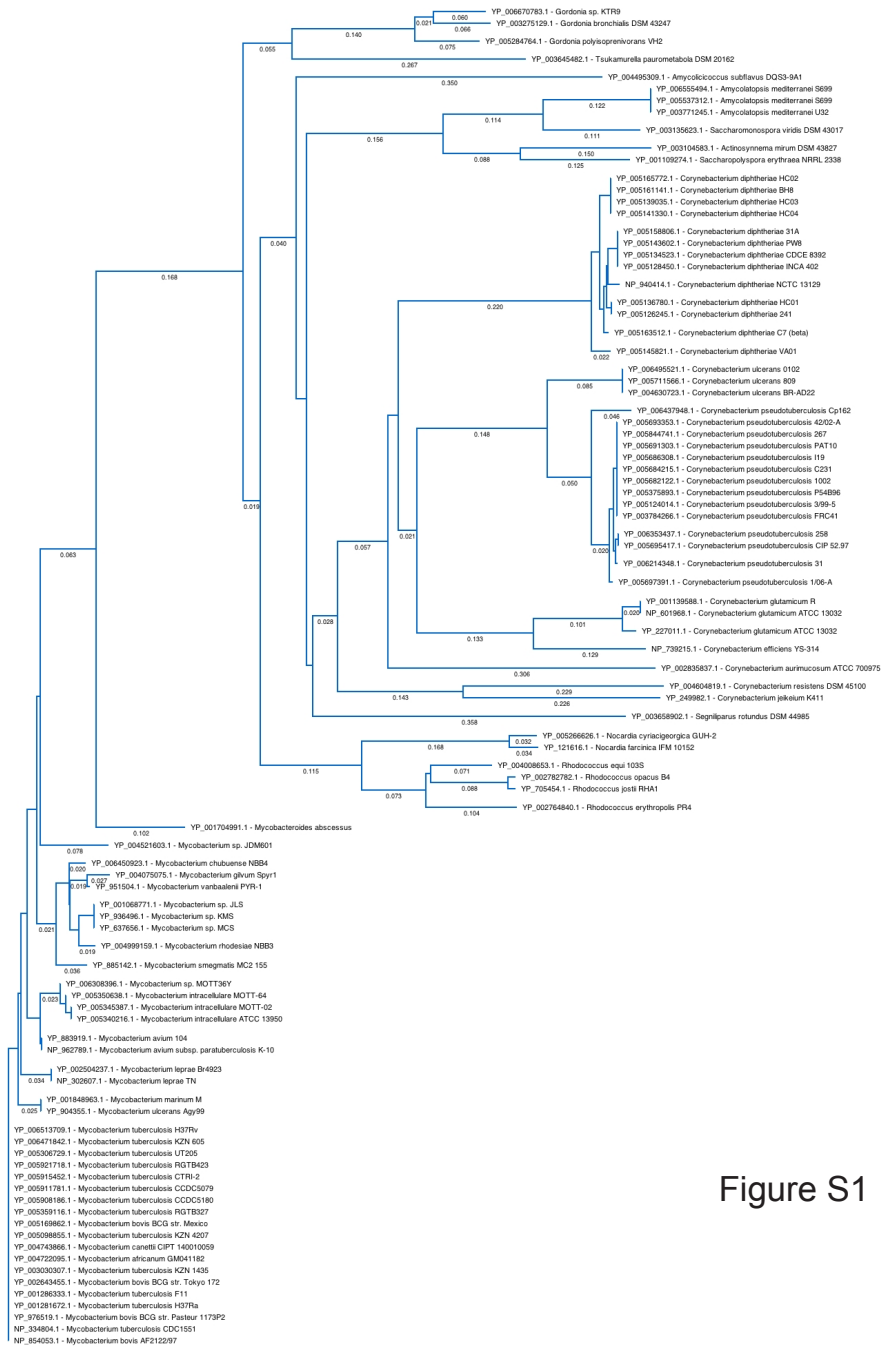

Figure S1

Supplement: FIG S1 [file mBio.00850-19-sf001.pdf]

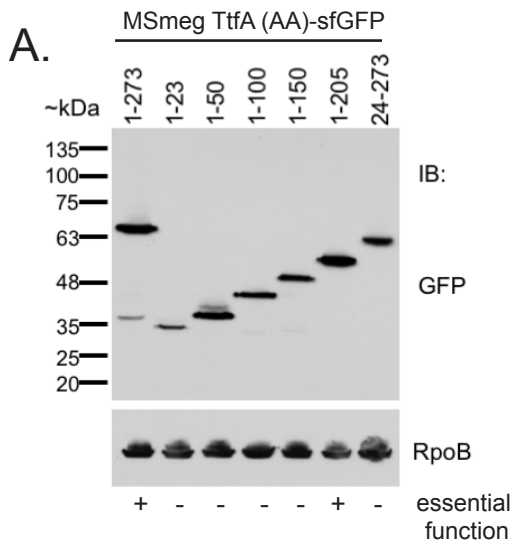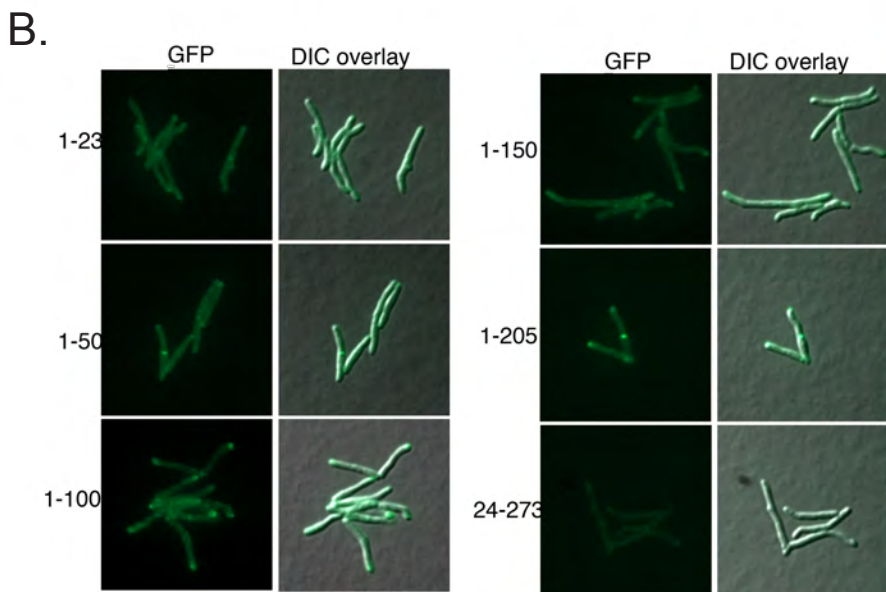

Figure S2

Supplement: FIG S2 [file mBio.00850-19-sf002.pdf]

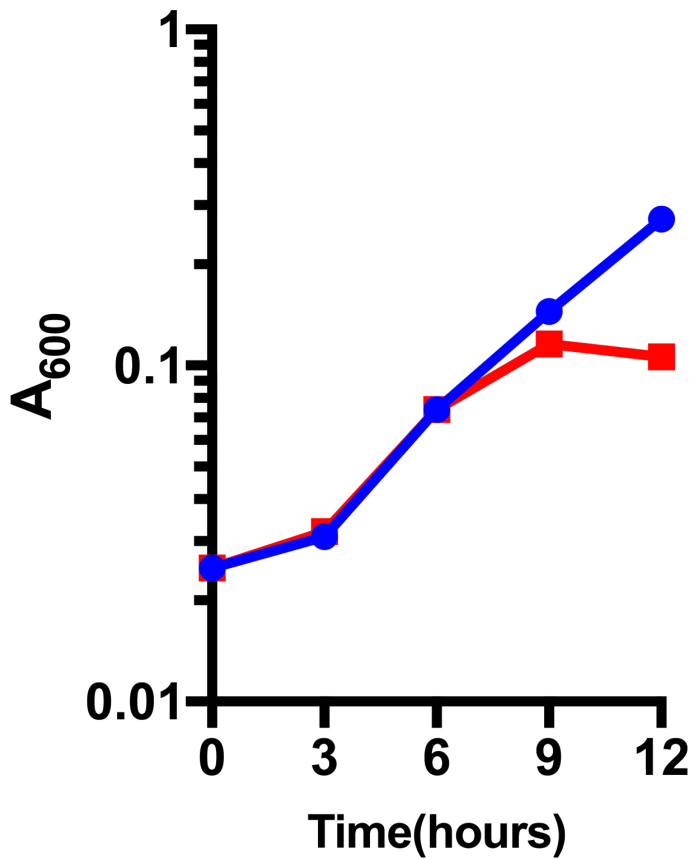

Figure S3

Supplement: FIG S3 [file mBio.00850-19-sf003.pdf]

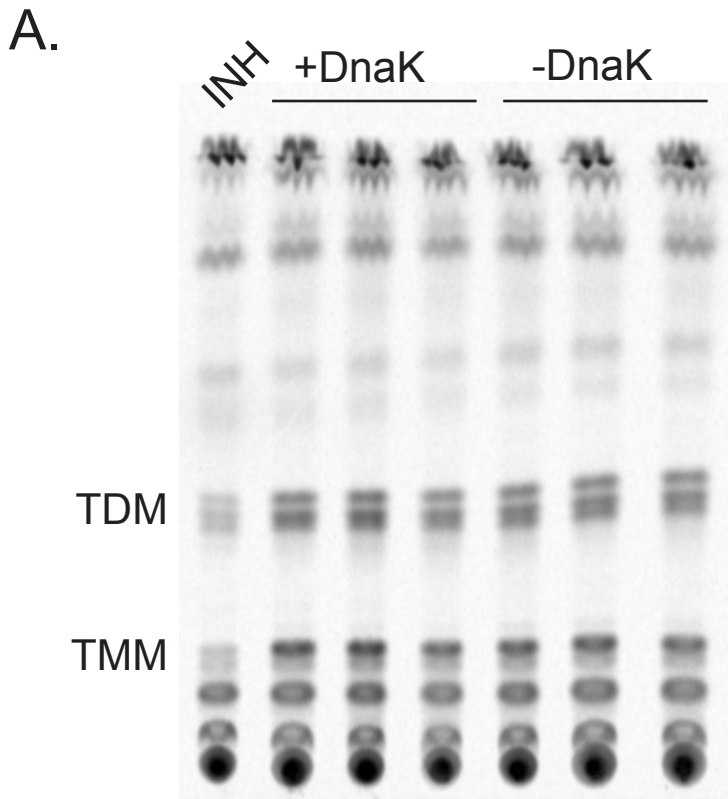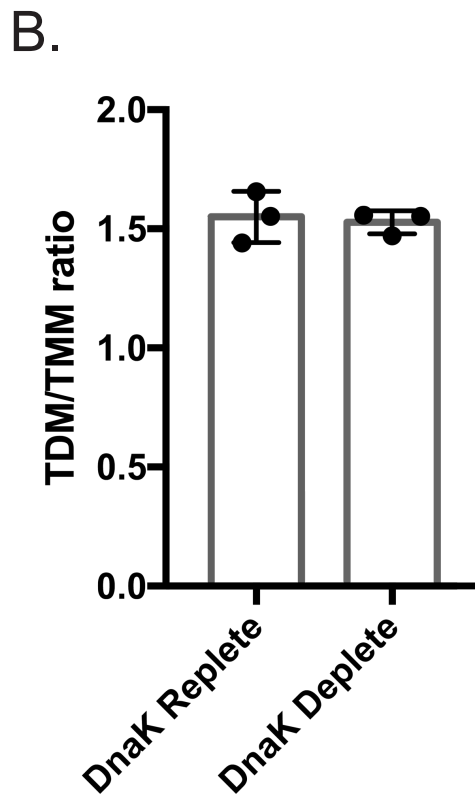

Figure S4

Supplement: FIG S4 [file mBio.00850-19-sf004.pdf]

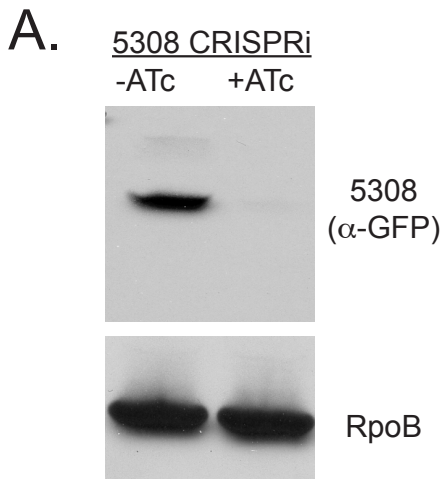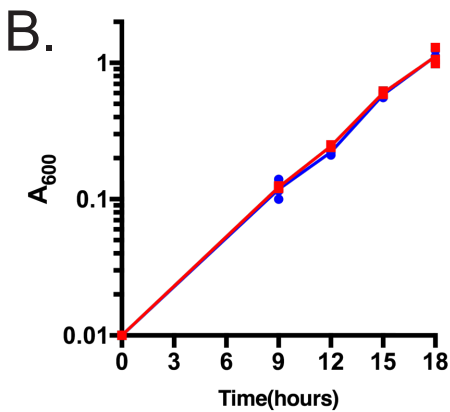

Figure S5

Supplement: FIG S5 [file mBio.00850-19-sf005.pdf]
